# Supplementary material for: Causal Relationship between Aging and Anorexia Nervosa: A White-Matter-Microstructure-Mediated Mendelian Randomization Analysis
Source: Biomedicines. 2024 Aug 16;12(8):1874. doi: 10.3390/biomedicines12081874 (PMC11351342; doi:10.3390/biomedicines12081874)
Supplement: Supplementary file 1 [file biomedicines-12-01874-s001.zip › Supplementary Figure/Figure S4/Singe SNP plot of MR analysis for TL to ubm-b-1536.pdf]

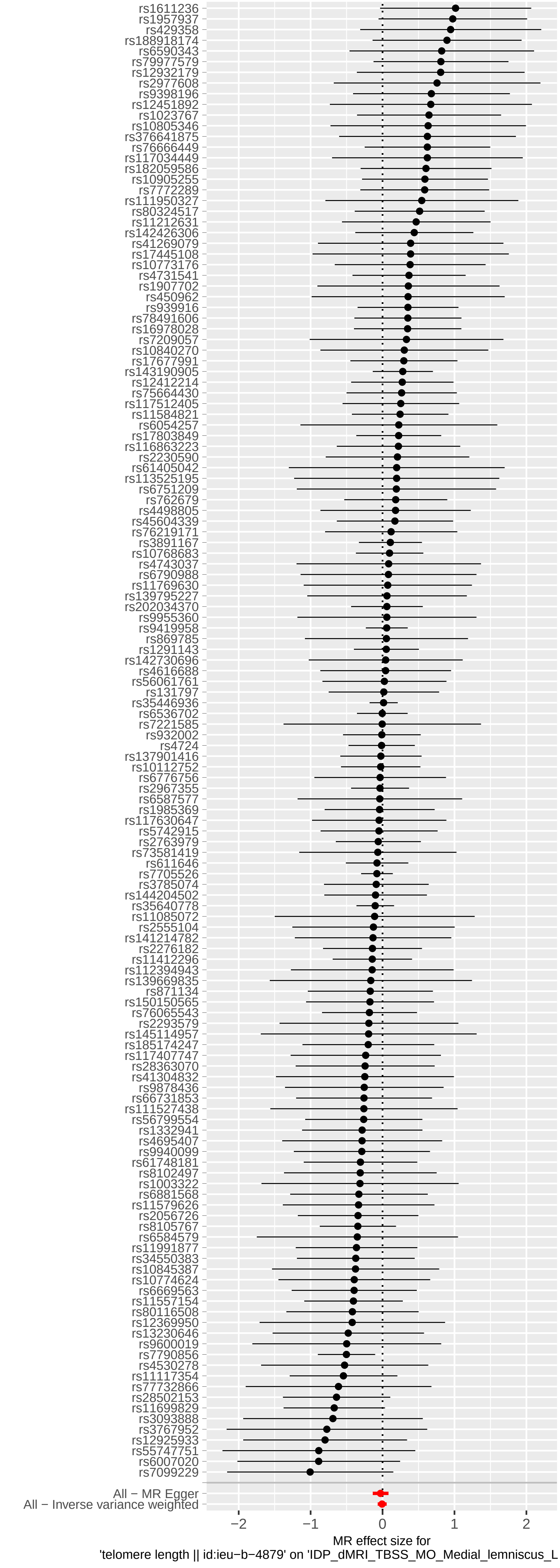
rs1611236
rs1957937
rs429358
rs188918174
rs6590343
rs79977579
rs12932179
rs2977608
rs9398196
rs12451892
rs1023767
rs10805346
rs376641875
rs76666449
rs117034449
rs182059586
rs10905255
rs7772289
rs111950327
rs80324517
rs11212631
rs142426306
rs41269079
rs17445108
rs10773176
rs4731541
rs1907702
rs450962
rs939916
rs78491606
rs16978028
rs7209057
rs10840270
rs17677991
rs143190905
rs12412214
rs75664430
rs117512405
rs11584821
rs6054257
rs17803849
rs116863223
rs2230590
rs61405042
rs113525195
rs6751209
rs762679
rs4498805
rs45604339
rs76219171
rs3891167
rs10768683
rs4743037
rs6790988
rs11769630
rs139795227
rs202034370
rs9955360
rs9419958
rs869785
rs1291143
rs142730696
rs4616688
rs56061761
rs131797
rs35446936
rs6536702
rs7221585
rs932002
rs4724
rs137901416
rs10112752
rs6776756
rs2967355
rs6587577
rs1985369
rs117630647
rs5742915
rs2763979
rs73581419
rs611646
rs7705526
rs3785074
rs144204502
rs35640778
rs11085072
rs2555104
rs141214782
rs2276182
rs11412296
rs112394943
rs139669835
rs871134
rs150150565
rs76065543
rs2293579
rs145114957
rs185174247
rs117407747
rs28363070
rs41304832
rs9878436
rs66731853
rs111527438
rs56799554
rs1332941
rs4695407
rs9940099
rs61748181
rs8102497
rs1003322
rs6881568
rs11579626
rs2056726
rs8105767
rs6584579
rs11991877
rs34550383
rs10845387
rs10774624
rs6669563
rs11557154
rs80116508
rs12369950
rs13230646
rs9600019
rs7790856
rs4530278
rs11117354
rs77732866
rs28502153
rs11699829
rs3093888
rs3767952
rs12925933
rs55747751
rs6007020
rs7099229
All − MR Egger
All − Inverse variance weighted
−2
−1
0
1
2
MR effect size for
'telomere length || id:ieu−b−4879' on 'IDP_dMRI_TBSS_MO_Medial_lemniscus_L |
